# Structure and non-essential function of glycerol kinase in *Plasmodium falciparum* blood stages

**DOI:** 10.1111/j.1365-2958.2008.06544.x

**Published:** 2009-01

**Authors:** Claudia Schnick, Spencer D Polley, Quinton L Fivelman, Lisa C Ranford-Cartwright, Shane R Wilkinson, James A Brannigan, Anthony J Wilkinson, David A Baker

**Affiliations:** 1 Structural Biology Laboratory, Department of Chemistry, University of YorkYork YO10 5YW, UK; 2 Department of Infectious and Tropical Diseases, London School of Hygiene and Tropical MedicineLondon, WC1E 7HT, UK; 3 Institute of Biomedical and Life Sciences, Division of Infection and Immunity, University of GlasgowGlasgow, G12 8TA, UK

## Abstract

Malaria pathology is caused by multiplication of asexual parasites within erythrocytes, whereas mosquito transmission of malaria is mediated by sexual precursor cells (gametocytes). Microarray analysis identified glycerol kinase (GK) as the second most highly upregulated gene in *Plasmodium falciparum* gametocytes with no expression detectable in asexual blood stage parasites. Phosphorylation of glycerol by GK is the rate-limiting step in glycerol utilization. Deletion of this gene from *P. falciparum* had no effect on asexual parasite growth, but surprisingly also had no effect on gametocyte development or exflagellation, suggesting that these life cycle stages do not utilize host-derived glycerol as a carbon source. Kinetic studies of purified PfGK showed that the enzyme is not regulated by fructose 1,6 bisphosphate. The high-resolution crystal structure of *P. falciparum* GK, the first of a eukaryotic GK, reveals two domains embracing a capacious ligand-binding groove. In the complexes of PfGK with glycerol and ADP, we observed closed and open forms of the active site respectively. The 27° domain opening is larger than in orthologous systems and exposes an extensive surface with potential for exploitation in selective inhibitor design should the enzyme prove to be essential *in vivo* either in the human or in the mosquito.

## Introduction

Malaria remains a major challenge to global health with 40% of the world population at risk. The burden of disease falls mainly on tropical Africa, accounting for more than 90% of the estimated 500 million annual cases (Greenwood *et al*., 2005). The disease is caused by the protozoan parasite *Plasmodium,* which is transmitted by the bite of a mosquito; the vast majority of deaths are due to infection with *Plasmodium falciparum*. The rapid spread of drug-resistant malaria parasites has led to an urgent need for new drugs. The life cycle of the parasite is complex. When an infectious mosquito takes a human blood meal, sporozoites released from its salivary glands enter the bloodstream and invade liver cells. Subsequently, thousands of merozoites are released which invade red blood cells where they replicate asexually and cause disease pathology. A small proportion of these merozoites differentiate into male and female gametocytes that form extracellular gametes on entering the mosquito midgut. Following fertilization, motile zygotes (ookinetes) develop into oocysts that contain thousands of sporozoites, which then migrate to the mosquito salivary glands. These are then passed on to another individual when the mosquito takes its next blood meal.

To better understand the biology of the sexual stage of the malaria parasite, we used *P. falciparum* whole genome microarrays to define a set of 246 genes in which transcription was gametocyte-specific (Young *et al*., 2005). One of the most highly upregulated transcripts encodes a putative glycerol kinase (GK; ATP:glycerol-3-phosphotransferase, EC 2.7.1.30). GK catalyses the rate-limiting step of glycerol utilization, with phosphorylation serving to sequester the sugar in the cell. The GK reaction is situated at a junction in metabolism. Following its phosphorylation, glycerol can be converted to dihydroxyacetone phosphate by glycerol-3-phosphate (G3P) dehydrogenase and fed into the glycolysis or gluconeogenesis pathways according to the metabolic status of the cell. Alternatively, it can serve as a precursor of glycerolipid biosynthesis by becoming fatty acid acylated to produce 1-acylglycerol 3 phosphate in a reaction catalysed by glycerol phosphate acyl transferase (Santiago *et al*., 2004). The latter role is likely to be especially important following infection of red blood cells by *P. falciparum* when the parasite grows prolifically and divides to produce up to 32 daughter cells over a 2 day period. This rapid growth is associated with active membrane biogenesis requiring *de novo* biosynthesis of the glycerolipids, phosphotidyl-ethanolamine and phosphatidyl-choline.

Glucose is the main source of energy for the parasite during malaria infection. Although glycerol phosphate can be derived from glucose, it would seem more efficient to utilize glycerol from the host serum for lipid biosynthesis to avoid utilization of the limiting substrate for growth. Indeed, glycerol from the host serum is incorporated into the membranes in some *Plasmodium* species (Holz, 1977; Vial and Ancelin, 1992). Red blood cells can take up this triose efficiently through the aquaglyceroporin AQP3 (Roudier *et al*., 1998) and it is presumed that a similar facilitator exists to allow passage of the substrate from the red cell cytoplasm into the parasitophorous vacuole. The *P. falciparum* genome (http://plasmodb.org/plasmo/) encodes a single aquaglyceroporin-like polypeptide that presumably facilitates entry of glycerol into the parasite.

Here we have characterized *P. falciparum* GK activity both *in vivo* and *in vitro* and present evidence that blood stage malaria parasites (asexual or sexual) do not utilize host-derived glycerol. To provide a platform for understanding substrate binding, catalysis and regulation in PfGK, we also determined its three-dimensional structure to reveal a dimer in which extensive domain motions accompany ligand binding.

## Results

### PfGK mRNA expression is upregulated in sexual blood stage parasites

**Fig. 1:**
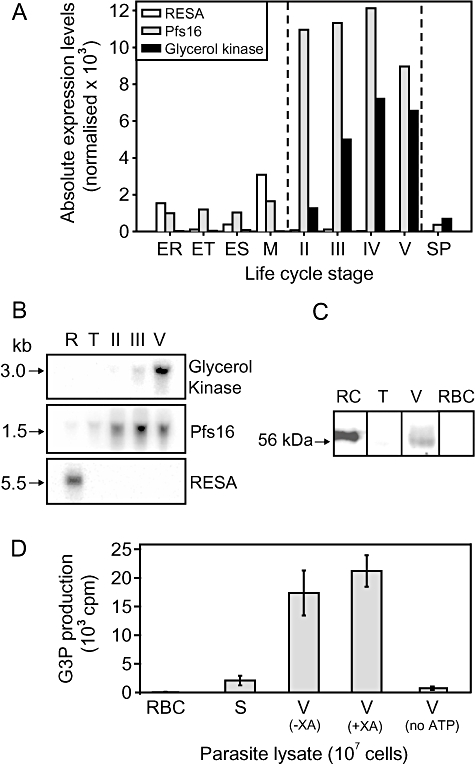
Sexual stage-specific expression of PfGK. A. Reanalysis of data obtained from screening of a *P. falciparum* full-genome high-density oligonucleotide array with cDNA derived from *P. falciparum* life cycle stage-specific mRNA (Young *et al*., 2005). The normalized absolute expression levels are presented for PfGK (PF13_0269), Pfs16 (a gametocyte-specific control, PFD0310w) and RESA (an asexual ring stage control, PFA0110w). ER, early rings; ET, early trophozoites; ES, early schizonts; M, merozoites; SP, sprozoites; II, III, IV and V are the individual gametocyte stages. B. Northern analysis using a probe derived from the *PfGK* coding region (upper panel). The blot was re-hybridized with *Pfs16* (middle panel) and *RESA* (bottom panel) probes. Lanes contain equivalent amounts of mRNA from asexual ring (R) and trophozoite (T) stage parasites and from gametocyte stages II, III and V. Transcript sizes (kb) are indicated to the left. C. Western blot using a rabbit antipeptide antibody. Recombinant PfGK (RC) expressed as a 6-His fusion protein was used as a positive control. Lanes containing proteins from trophozoites (T) and stage V gametocytes (V) are shown. Proteins extracted from human red blood cells (RBC) were used as a negative control. Lanes separated by vertical lines indicate individual samples that were not adjacent to each other on the blot. The size of native PfGK is indicated to the left. D. Native GK enzyme activity presented as counts per minute (cpm). Lysates from gametocytes before (−Xa) and after (+Xa) stimulation of gametogenesis with xanthurenic acid; schizonts (S); human RBC and a negative control in which no ATP was added to the gametocyte GK activity assay.

A *P. falciparum* full-genome high-density oligonucleotide microarray was hybridized with cDNA derived from cultures of highly synchronous asexual and sexual blood stage parasites. A potential GK orthologue, *PfGK* (PlasmoDB identifier: PF13_0269) was one of the most highly upregulated genes in gametocytes, but expression levels were barely detectable in asexual stage parasites (Fig. 1A). Northern blot analysis confirmed these findings; *PfGK* transcripts were detectable from early (stage II) to mature (stage V) gametocytes, but were not detectable in asexual ring or trophozoite stage parasites (Fig. 1B). A PfGK antipeptide antibody reacted strongly with a band of ∼56 kDa in Western blots containing mature gametocyte proteins. Little or no signal could be detected in asexual blood stage protein preparations, adding to the evidence that GK expression is either largely or exclusively sexual stage-specific (Fig. 1C). Measurement of GK activity in cell lysates from either stage V gametocytes or purified schizonts demonstrated that enzyme activity was confined to sexual stage parasites (Fig. 1D). To determine whether stimulation of gametogenesis caused an increase in GK activity, we added xanthurenic acid to mature gametocytes, but no significant increase was observed. The expression profile of PfGK was confirmed by utilizing the 5′ upstream sequence of *PfGK* to drive expression of green fluorescent protein (GFP) in episomally transformed parasites. The resulting transfectants showed evidence of GK promoter activity in both male and female gametocytes, but not in asexual blood stage parasites (Fig. S1).

### PfGK is a functional GK

To characterize PfGK biochemically, the deduced 501-amino-acid-residue coding sequence was expressed as a fusion protein with maltose-binding protein (MBP) in *E. coli*. GK-deficient *E. coli* mutants were transformed with a plasmid expressing the PfGK–MBP fusion protein and plated on McConkey agar plates containing glycerol. Mutants transformed with empty vector plasmid produced yellow colonies, indicating that they were unable to utilize glycerol as a carbon source, whereas transformation with the plasmid containing the *PfGK–MBP* gene produced bright pink/purple colonies. Thus PfGK can functionally complement the *E. coli* GK-deficient mutants (Fig. S2).

The PfGK fusion protein was purified before removal of the MBP portion and determination of K_M_ and V_max_ for both glycerol and ATP. For each substrate, the kinetic analysis was performed at a constant and saturating concentration of the partner substrate, this being 5 mM glycerol and 2.5 mM ATP (Fig. S3). The V_max_ values (U mg^−1^) were 15.5 ± 0.4 for glycerol and 18.3 ± 0.35 for ATP. The K_M_ values of 21 ± 1 μM (ATP) and 18 ± 2 μM (glycerol) are similar for both substrates and comparable to those determined for *E. coli* GK (EcGK) (Feese *et al*., 1998). The glycolytic intermediate fructose-1,6-bisphosphate (FBP) is an allosteric effector of EcGK that traps the enzyme in an inactive tetrameric state with a K_i_ of 0.25 mM (Ormo *et al*., 1998). At the highest concentrations of FBP tested (100 mM) the decrease of the rate of glycerol phosphorylation by PfGK was only twofold, suggesting that FBP is not a physiological regulator of PfGK (Fig. S4).

### GK is not required for asexual blood stage replication, gametocyte development or exflagellation

**Fig. 2:**
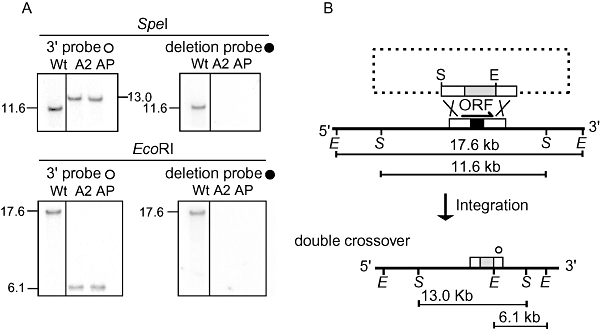
Schematic of the Wt and PfGK^-^ disrupted loci with restriction fragment sizes predicted and confirmed by Southern blotting. A. Southern blot analysis of *P. falciparum* PfGK^-^ clones and a Wt 3D7 clone. A2 and AP are independent PfGK^-^ clones (double-crossover recombination). Band sizes are indicated by arrows. Restriction digests with SpeI (above) and EcoRI (below) are shown. Blots were probed with two ^32^P-labelled probes. The first (3′ probe, indicated by a white circle) corresponds to the PfGK coding region immediately downstream of the deleted sequence and the second (deletion probe, indicated by a black circle) corresponds to the deleted sequence shown in black on the Wt schematic (right panels). B. Schematic diagram showing the gene knockout strategy using the pHTK-GK vector (dotted). The expected band sizes of the Wt and recombinant PfGK loci following integration are shown below. S and E denote SpeI and EcoRI sites respectively.

**Fig. 3:**
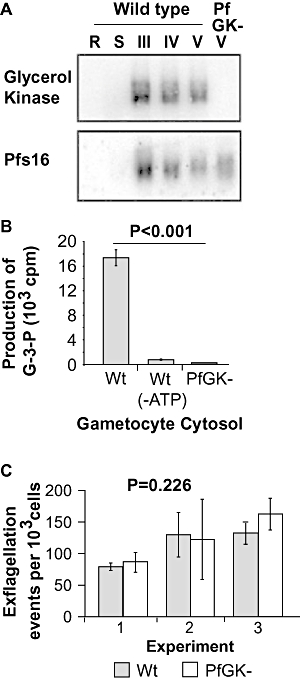
Phenotype analysis of GK knockout clones. A. Northern analysis of *P. falciparum* WT and PfGK^-^ (clone A22F) probed with the deleted portion (upper panel) and a Pfs16 probe. The blot contains Wt 3D7 mRNA samples from asexual ring (R) and schizont (S) stages and gametocyte stages III–V as well as mRNA from PfGK^-^ stage V gametocytes. B. Native GK enzyme activity is presented as counts per minute (cpm) incorporated in lysates from Wt and PfGK^-^ gametocytes. A negative control is included in which no ATP was added to the Wt enzyme assay. The error bars represent the 95% confidence interval of the mean enzyme activity from multiple assays, which ranged in number from 9 (PfGK^-^) to 13 (Wt) utilizing at least two different gametocyte preparations for both Wt and PfGK^-^ parasites. The *P*-value shows the significance of the difference between the activity levels of Wt and PfGK^-^ as calculated by a Mann–Whitney *U*-test in stata. C. Levels of exflagellation in *P. falciparum* Wt and a PfGK^-^ clone. The number of exflagellation events per 10^3^ cells was counted. The results are from three separate experiments counted in duplicate. The error bars represent the 95% confidence interval of the mean of the two readings within a single experiment, while the *P*-value was calculated by a Poisson regression in stata to look for a significant difference in exflagellation rates while adjusting for the effects of the different exflagellation rates in different experiments.

To investigate the role of GK in *P. falciparum* development, we disrupted the *PfGK* gene (Fig. 2) which is the only *GK* orthologue in the genome. Clones were obtained that had undergone double-crossover homologous recombination events, resulting in the deletion of the C-terminal 338 residues of PfGK. This region encompasses the majority of the ATP-binding motifs (see below), and therefore the truncated gene arising from the integration event is expected to be inactive. The genotype was verified by a combination of PCR and Southern blot analysis (Fig. 2). Northern blot analysis confirmed that the mutant no longer expressed *PfGK* mRNA (representative clone shown in Fig. 3A). Gametocytes from both PfGK^-^ and wild-type (Wt) parasites were tested for GK enzyme activity. GK enzyme activity was absent in the PfGK^-^ mutant (Fig. 3B), confirming functional disruption of the *PfGK* gene.

Disruption of the *PfGK* gene had no measurable effect on asexual blood stage growth (data not shown), consistent with the evidence that the gene is not expressed during this phase of the life cycle. Somewhat surprisingly, disruption of the gene had no detectable effect on gametocyte development, even though the enzyme is active in Wt gametocytes. Furthermore, levels of ‘rounding-up’ (the initial morphological change of gametogenesis) of male and female gametocytes as well as exflagellation (emergence of motile male gametes from red blood cells) (Fig. 3C) were equivalent in control and mutant clones.

### Structure solution and overall structure of PfGK

The crystal structure of PfGK in complex with glycerol was solved by molecular replacement using EcGK (Feese *et al*., 1998) as a search model. The structure was refined against data extending to 1.5 Å spacing to give a final model that agrees well with the X-ray terms (Table 1).

**Table 1:** Data collection and refinement statistics.

	PfGK–glycerol	PfGK–ADP
Data collection		
Space group	*P*2_1_	*P*2_1_
Unit cell dimensions		
*a*, *b*, *c* (Å)	101.9, 100.7, 107.8	82.5, 57.6, 123.9
α, β, γ (°)	90.0, 92.5, 90.0	90.0, 89.75, 90.0
Resolution (Å)	107.83–1.49 (1.53–1.49)^a^	124.03–2.41 (2.47–2.41)
*R*_sym_^b^	0.084 (0.744)	0.104 (0.843)
*I*/σ*I*	11.99 (2.23)	10.95 (0.71)
Completeness (%)	73.6 (18.4)^c^	91.4 (40.6)
Redundancy	4.3 (3.4)	3.7 (1.9)
Refinement		
Resolution (Å)	37.85–1.49	49.45–2.41
No. reflections	247 409 (4574)	39 658 (1280)
*R*-factor^d^/*R*_free_^e^	0.15/0.20	0.19/0.25
Asymmetric Unit	2 dimers	1 dimer
No. atoms		
Protein	16 116	8032
Ligand	72 glycerol 248 ethylene glycol	54 ADP 84 ethylene glycol
Water	1929	307
*B*-factors (Å^2^)		
Protein	15.5	45.6
Ligand	23.9	54.4
Water	30.5	44.8
Root mean squared deviation^f^		
Bond lengths (Å)	0.015	0.018
Bond angles (°)	1.66	1.76

a Values in parentheses are for highest-resolution shell.

b *R*_sym_ = Σ_*hkl*_Σ_*i*_|*I*_*i*_ − *<I*> |*/*Σ_*hkl*_Σ_*i*_*<I*> where *I*_*i*_ is the intensity of the *i*th measurement of a reflection with indexes *hkl* and *<I*> is the statistically weighted average reflection intensity.

c The completeness in the resolution shell 1.85–1.80 Å is 96% and in the resolution shell 1.72–1.67 Å it is 54%.

d *R*-factor = Σ||*F*_*o*_| − |*F*_*c*_||/Σ |*F*_*o*_| where *F*_*o*_ and *F*_*c*_ are the observed and calculated structure factor amplitudes respectively.

e *R*_free_ is the *R*-factor calculated with 5% of the reflections chosen at random and omitted from refinement.

f Root mean square deviation of bond lengths or bond angles from ideal geometry.

**Fig. 4:**
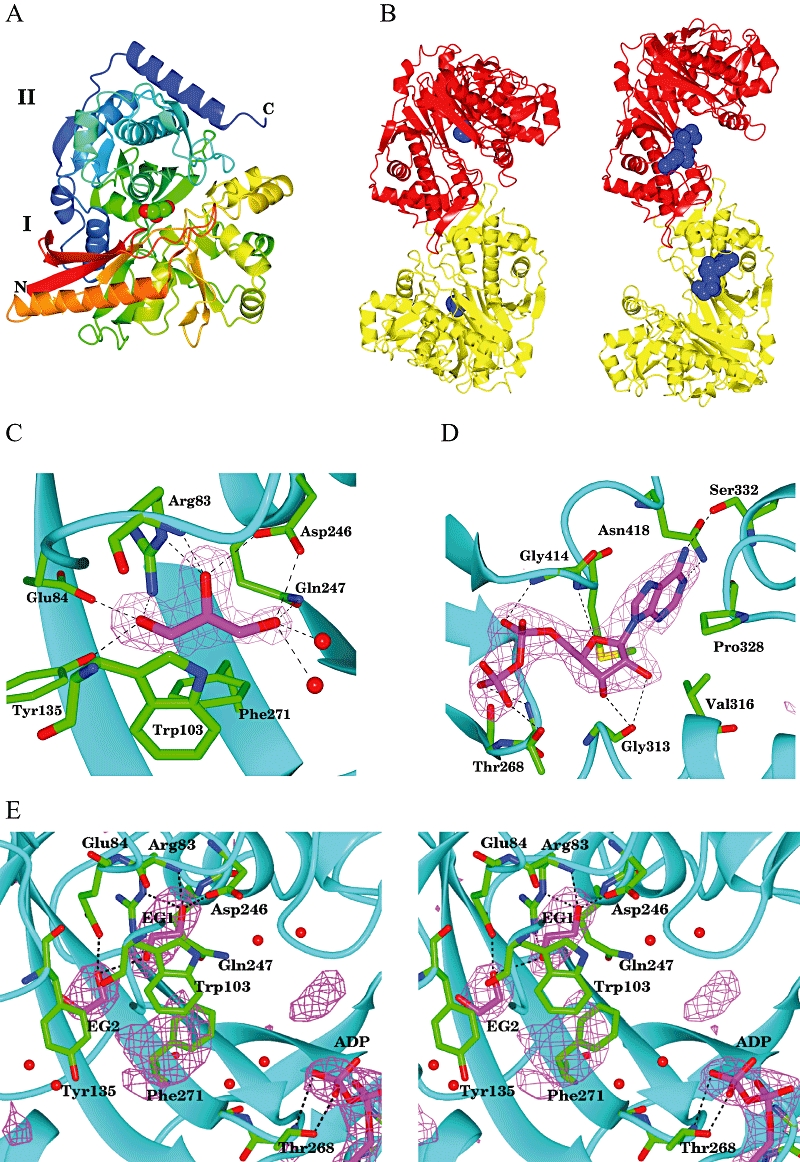
Protein structure. A. Ribbon tracing of PfGK–glycerol, colour ramped from the N-terminus (red) to the C-terminus (blue) and with the domains labelled I and II. The glycerol ligand is shown in space-filling representation coloured by atom. B. Ribbon tracing of the PfGK–glycerol and PfGK–ADP dimers with the chains coloured red and yellow. The glycerol and ADP ligands are shown in space-filling representation in closed (left) and open (right) forms of the structure respectively. The view is such that the dimer-forming domains (II) are in the same orientation in each structure. C. The glycerol-binding site in PfGK–glycerol with the glycerol ligand displayed with its associated electron density and with surrounding protein residues labelled. For map calculation, the glycerol ligand was deleted from the final co-ordinate set and five cycles of refinement in refmac were carried out. The map was calculated with sigma A weighted coefficients *F*_obs_ − *F*_calc_ and α_calc_ and is contoured at 4σ. Dashed lines indicate polar interactions and atoms are coloured according to type: C green (except for glycerol), O red and N blue. Two water molecules that bond to the C3-OH are shown as red spheres. D. The ADP binding site in the PfGK–ADP complex with the ADP ligand displayed with its associated electron density. An omit map was calculated as in (C) and the electron density is displayed at the 3σ level. E. Stereo-view of the glycerol-binding site in the PfGK–ADP complex. Residues that form polar interactions with the glycerol in the PfGK–glycerol complex are shown surrounding a pair of ethylene glycol molecules (EG1 and EG2). The phosphate groups of the ADP can be seen in the lower right hand corner of the image. The electron density displayed is an omit map calculated after deletion of the ADP, EG1 and EG2 species from the co-ordinate set and following five cycles of refmac refinement. The map is displayed at the 3σ level. It is apparent that although the glycerol-binding pocket is rearranged, the pair of ethylene glycol molecules together mimic many of the interactions formed by the glycerol. It is also apparent that there are additional electron density features in the cavity of the open structure that we have been unable to account for in our model, including a flat section stacked between the aromatic rings of Trp103 and Phe271 in both subunits of the dimer.

**Fig. 5:**
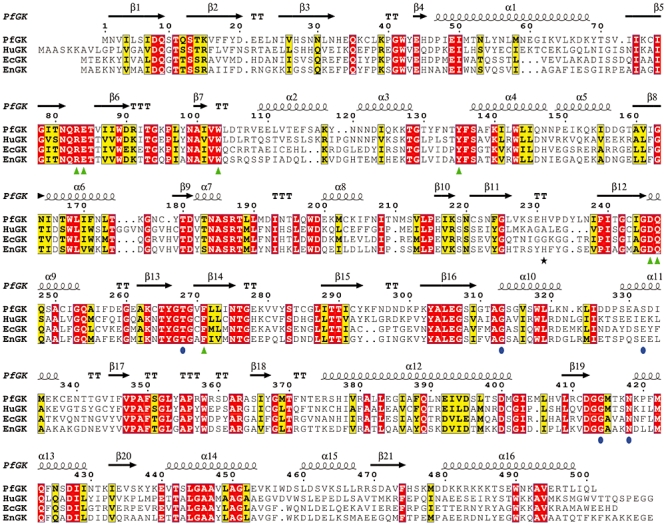
Protein sequence alignment of GK from *P. falciparum*, human, *E. coli* and *En. casseliflavus*. This figure was generated with ESPript (Gouet *et al*., 1999) and adjusted manually to optimize structure-based alignment. The secondary structure elements and turns (TT) of PfGK are shown. Strictly conserved residues are boxed and coloured red. Residues implicated in ligand binding are indicated with green triangles (glycerol) and blue circles (ADP). The phosphorylated histidine of EnGK is marked with a star. For clarity, the C-terminal 31 residues of Human GK are not shown.

The 501-residue GK polypeptide is arranged as two domains separated by a deep cleft (Fig. 4). Each domain is constructed around a βββαβαβα core that is characteristic of the sugar kinase/Hsp70/actin superfamily of proteins (Bork *et al*., 1992; Hurley, 1996). Domain I (residues 1–262 and 436–471) comprises β_3_β_2_β_1_α_1_β_5_α_6_β_12_α_9_ and domain II (residues 263–435 and 472–501) consists of β_16_β_14_β_13_α_12_β_19_α_13_β_20_α_14_ (Fig. 5). Elaborations of this core endow GK with distinct features. In domain I, large insertions before and after helix α_6_ produce a compact, largely α-helical subdomain. In domain II, strands β_15_ and β_21_ flank the core β-sheet and extend it to seven strands. The principal insertions are between β_16_ and α_12_, and at the C-terminus where the 50 residues following helix α_14_ form a strap around the periphery of domain II. In agreement with gel filtration experiments (data not shown), PfGK is a dimer in the crystal (Fig. 4B). The majority of the dimer-forming segments of the polypeptide are in domain II. At the heart of the interface, strands β_17_ from each chain align in an antiparallel manner so that an intermolecular four-stranded β-sheet is formed. In the dimer, 1750 Å^2^ of otherwise accessible surface area on each subunit is buried.

### Ligand binding and domain movement

**Fig. 6:**
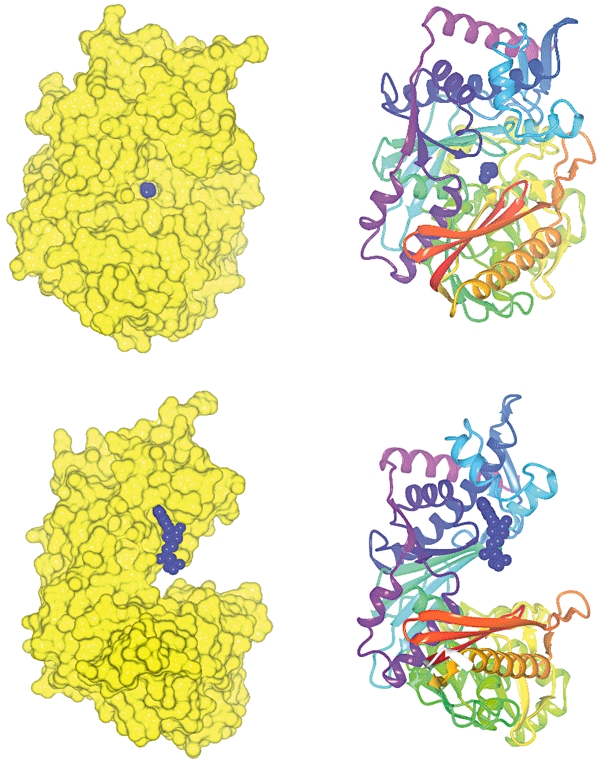
Structure representation of protein with bound ligands. (Left) Surface rendering and (Right) ribbon diagram of a PfGK subunit with bound ligands glycerol (top) and ADP (bottom) shown in space-filling representation (blue).

Glycerol is defined by electron density deep in the cavity between the domains (Figs 4 and 6). Its hydroxyl groups fulfil their hydrogen bonding potential through charge–dipole interactions with Glu84, Arg83 and Asp246 and dipole–dipole interactions with Arg83, Tyr135 and Gln247 (Fig. 4C). The C3-OH of the glycerol forms hydrogen bonds to two water molecules that occupy a narrow channel to the protein surface. The apolar face of the glycerol moiety packs against the side-chains of Trp103 and Phe271 (Fig. 4C), the latter representing the only glycerol-binding residue contributed by domain II. The pattern of interactions is similar to that in glycerol complexes of bacterial GKs (Hurley *et al*., 1993; Yeh *et al*., 2004). The PfGK–ADP crystals have different cell parameters (Table 1) and it is apparent that domains I and II have moved apart and that the enzyme is in an ‘open’ conformation (Fig. 4B). The relative domain movement can be described as a 27.5° rotation, substantially larger than the 7° rotation deduced from crystal structures of orthologous enzymes (Bystrom *et al*., 1999; Yeh *et al*., 2004). In the dimer, the long axis increases in length from 97 Å to 117 Å (Movie S1), exposing a further 1500 Å^2^ of solvent accessible surface area around the active site. In the open (ADP-bound) structure, interdomain contacts such as Gln12-Thr268 and the salt-bridges Arg83-Glu306 and Glu109-Arg363 are ruptured. The largest changes in dihedral angles occur at residues Phe271-Leu272 and Glu306-Ser308 near the glycerol-binding site and Ser470-Asp471. Aspects of the glycerol pocket are maintained, with a pair of ethylene glycol (cryoprotectant) molecules clearly mimicking protein interactions formed by glycerol (Fig. 4E). However, as a result of domain movement, Phe271 and Tyr135 are significantly displaced (Fig. 4C and E). Residue Tyr354, whose hydrophobic nature is conserved among eukaryotic GK sequences, occludes the alternate conformer of Tyr135 in the closed form of the enzyme.

In contrast to glycerol, which binds predominantly to domain I, ADP in the PfGK–ADP complex is bound exclusively to domain II (Fig. 4B). ADP is bound with the adenine base tucked into a pocket and the ribose and phosphate moieties more exposed on the enzyme surface extending towards the interdomain cleft. The adenine base forms polar interactions with Asn418 and Ser332. The rather modest set of polar interactions with the ribose and phosphate groups involving Thr268, Gly313, Gly414 and Met415 (Fig. 4D) are likely to be augmented in the ternary closed complex with ATP. The glycerol and adenine nucleotide binding sites are sufficiently similar to that of EcGK to infer a common phosphotransfer mechanism of action in which the binding of glycerol and ATP to domains I and II respectively, is followed by domain closure to sequester the phosphotransfer chemistry from the bulk solvent. A plausible mechanism foresees the base catalysed activation of the C3 hydroxyl of the glycerol by Asp246 for in-line nucleophilic attack on the γ-phosphorus of the nucleotide (Hurley *et al*., 1993). The accumulating charge in the pentaco-ordinate phosphate intermediate would be stabilized by surrounding protein residues prior to its collapse to form the products G3P and ADP.

## Discussion

### Glycerol metabolism in *P. falciparum*

Glycerol kinase is a ubiquitous enzyme with important roles in carbohydrate and lipid metabolism in plants and animals. Interference in the function of GK can have adverse consequences in many organisms, ranging from reduced growth on glycerol in fungi (Witteveen *et al*., 1990) to debilitating disease in humans (McCabe, 2001). In this study, we have shown that disruption of the malaria parasite GK gene has no effect on the blood stages of the parasite life cycle even though these stages are characterized by high-energy requirements and prolific metabolic activity, including glycerolipid biosynthesis. It is known that there is a functional aquaglyceroporin in *P. falciparum* and it is possible that glycerol is acquired by the parasite from the human host serum via the erythrocyte cytoplasm. It has therefore been tempting to speculate that serum glycerol is a source of G3P for the parasite (Hansen *et al*., 2002). However, it is clear from our results that synthesis of G3P by the parasite GK does not occur during the growth of *P. falciparum* asexual blood stages *in vitro*. This suggests that under these conditions, energy production and/or glycerolipid biosynthesis in these life cycle stages occurs independently of the parasite GK and host-derived glycerol. It is therefore likely that glycolytic processing of glucose is the source of G3P in asexual blood stage parasites.

It has been shown in *Plasmodium berghei* that many mRNA species are stored, and remain untranslated in female gametocytes until the parasites are later taken up by a mosquito. Deletion of the gene encoding an RNA helicase, Dozi (development of zygote inhibited) abrogated this translational repression for more than 370 transcripts (Mair *et al*., 2006). In the absence of sex-specific differences in regulation, it is likely that the phenomenon of translational repression does not explain the lack of phenotype in PfGK^-^ gametocytes because GK protein and enzyme activity were detectable in Wt (but not PfGK^-^) gametocytes. Even so, the mutant gametocytes developed normally and male gametocytes exflagellated. This suggests that while GK can phosphorylate glycerol in Wt gametocytes, the resulting intracellular pool of G3P (or its subsequent metabolic products) is not utilized until later in development within the mosquito.

### GK *in vitro* and *in vivo*

The non-essential nature of GK in *P. falciparum* asexual replication is surprising as it is known that host glycerol is taken up by *Plasmodium knowlesi* and incorporated into parasite phospholipids (Vial and Ancelin, 1992). In contrast to *P. falciparum*, the GK gene from the rodent malaria parasite *P. berghei* is refractory to deletion (C. Janse, A. Waters, Leiden, Netherlands; D.A.B., unpubl. obs.), suggesting that *PbGK* may be essential for asexual blood stage growth in this species. This finding is consistent with the detection of PbGK mRNA in asexual blood stages (A. Waters, pers. comm.) and our measurements of GK activity in *P. berghei* asexual blood stage parasite extracts (data not shown). Mutants of *P. berghei* in which the aquaglyceroporin orthologue has been deleted are viable, but these parasites are defective in glycerol transport and lower levels of asexual multiplication occur relative to Wt parasites (Promeneur *et al*., 2007). Transgenic mice lacking the aquaglyceroporin AQP9 (defective in the rapid uptake of glycerol) can support growth of *P. berghei*, but the lethal effects of infection are delayed (Liu *et al*., 2007), suggesting that uptake of glycerol plays a role in virulence in *P. berghei* infection. In contrast, our results show that utilization of exogenous glycerol is not important in *P. falciparum* blood stage parasites *in vitro*, highlighting a possible difference in metabolism between these two *Plasmodium* species. Alternatively this may reflect the plentiful supply of glucose available to *P. falciparum* grown *in vitro*.

A recent paper has examined gene expression using a full-genome microarray with *P. falciparum* mRNA isolated directly from patients (Daily *et al*., 2007). By comparing the patterns of expression with those of yeast grown under different conditions, three distinct clusters of malaria parasites were defined. In terms of genes/pathways turned on and off, the clusters resembled: yeast grown under normal conditions, under starvation conditions and yeast exposed to environmental stress. Intriguingly in the context of the present study, GK (and other genes implicated in glycerol metabolism) is upregulated in the cluster corresponding to yeast grown under starvation conditions. It seems therefore that in certain clinical situations, GK may be expressed in *P. falciparum* asexual blood stage parasites *in vivo*. For example, patients often become hypoglycaemic and, if glucose became limiting, then exogenous glycerol might be utilized as previously suggested (Beitz, 2007). It is also possible that host-derived glycerol is not utilized (and PfGK not expressed) by *P. falciparum* blood stages *in vivo* and that the PfGK transcripts detected in malaria patient samples (Daily *et al*., 2007) reflect the presence of circulating gametocytes and sexually committed ring stage parasites.

### Implications for regulation of PfGK

As an enzyme that catalyses a reaction that sits at an important junction in metabolism, GK is normally subject to metabolic regulation. In contrast to the common catalytic mechanism suggested by crystal structures and sequence comparisons, the regulatory mechanisms differ between species. GK from *E. coli* is allosterically regulated by the glycolytic intermediate fructose (1,6)-bisphosphate (FBP). FBP promotes the assembly of active enzyme dimers into inactive tetramer forms. The crystal structure of the EcGK: FBP complex reveals a stoichiometry of two FBP molecules per GK tetramer with each FBP species bound to a pair of symmetry related glycine-rich loops (I^229^GGKGGTR^236^) situated at the dimer–dimer interface in the tetramer (Fig. S4), some 25 Å from the active site (Ormo *et al*., 1998). The glycine residues form polar interactions with the phosphate groups of the sugar, which additionally form ion-pairing interactions with the side-chains of the two Arg236 residues which are critical for regulation. The data presented here show that PfGK is not inhibited by FBP at concentrations that are likely to be encountered physiologically. Moreover, as is evident from Fig. 5 and Fig. S4, the FBP binding site in EcGK is altered in PfGK. In PfGK, the β_11_-β_12_ loop (residues 227–238, Fig. 5) corresponding to the glycine-rich loop in EcGK is well defined in the electron density maps. In contrast to the exposure of these loops in EcGK, in PfGK this loop is oriented towards the core of the dimer, an orientation that would sterically hinder tetramer formation. Moreover, the loop is devoid of the glycine and arginine residues (Fig. 5) that mediate FBP binding in EcGK, supporting the conclusion that PfGK is not regulated by FBP.

EcGK is also allosterically regulated by the phosphoenolpyruvate: phosphotransferase system (PTS) protein IIA^Glc^ which serves to ensure that glycerol is not utilized when more rapidly metabolized sources of energy are present. IIA^Glc^, in its unphosphorylated state binds to and inhibits EcGK. The structure of the EcGK: IIA^Glc^ complex reveals that the IIA^Glc^ inhibitor binds to a helical segment of domain II (residues 474–481), 30 Å from and distal to the active site (Hurley *et al*., 1993). As the phosphorylatable histidine is at the heart of the EcGK binding surface, it is clear why phosphorylation of IIA^Glc^, which occurs in the absence of PTS substrates, prevents GK binding and inhibition. A blast search of the *P. falciparum* genome for proteins with similarity to IIA^Glc^ identified no obvious candidates, indicating that this form of regulation is not important in this organism. It is interesting, nevertheless, that the sequence of the amino terminus of helix α16 of PfGK, which corresponds to the IIA^Glc^ binding site in EcGK, features a string of six consecutive basic residues (Fig. 5).

The PTS regulation of GK activity also occurs in Gram-positive bacteria. In these organisms, the modification is covalent rather than allosteric, with phosphorylation of a conserved histidine on GK by the PTS component HPr leading to enzyme activation (Darbon *et al*., 1999). This histidine is situated in the loop corresponding to the glycine-rich loop of EcGK and the crystal structure of GK from *Enterococcus casseliflavus* shows that this residue is exposed on the enzyme surface (Yeh *et al*., 2004). Surprisingly however, the structure shows a quite different arrangement of the subunits in the dimer such that the two phosphorylatable histidines (His232) in the EnGK are adjacent. This histidine residue is conserved in PfGK (Fig. 5), although it remains to be determined whether PfGK is regulated by phosphorylation.

The common theme in bacterial regulation of GK activity is long-range communication between the site of effector binding/modification and the site of ligand binding and catalysis. This suggests that regulatory sites in PfGK will not be discernible in the crystal structure in the absence of further experimental information.

## Conclusions

The non-essential character of GK in the blood stages of *P. falciparum* infection *in vitro* is surprising given the extent of upregulation of its gene, and the demonstration of enzyme activity in the sexual red blood stages (gametocytes). The results may mask an essential function *in vivo*, alternatively the upregulation in the sexual stages may anticipate an essential role in the mosquito stages which have not been investigated. The three-dimensional structure of PfGK which is the first, to our knowledge, of a eukaryotic GK, suggests a common catalytic mechanism with the bacterial GKs and indicates that these enzymes can undergo very large conformation changes in their reaction cycle. The extensive surface of PfGK which embraces the ligand binding site could be exploited for the design of specific inhibitors should the enzyme prove to have an essential role in the parasite life cycle *in vivo*.

## Experimental procedures

### Parasite cultivation

Gametocyte-producing *P. falciparum* clone 3D7 was maintained in asexual form in A+ red blood cells in 10% serum under standard conditions (Trager and Jensen, 1976). Stage V gametocytes were Nycodenz-purified as previously described (Fivelman *et al*., 2007) and gametogenesis was induced with 20 μM xanthurenic acid (Billker *et al*., 1998). For exflagellation assays, cells were resuspended at a concentration of 5 × 10^6^ ml^−1^. Ten minutes after induction, cells were observed by light microscopy and counted for a further 10 min, during which time up to 10 000 cells were observed.

### Generation and genotyping of transgenic parasites and phenotype analysis

Genetic manipulation of 3D7 was achieved by transfection with pHTK-based plasmids (Duraisingh *et al*., 2002). Transfection of *P. falciparum* 3D7 parasites was carried out according to standard procedures (Deitsch *et al*., 2001) and drug selection performed using WR99210 and ganciclovir (Duraisingh *et al*., 2002). Parasite lines were cloned by limiting dilution. Genotyping was carried out by a combination of PCR using primer pairs diagnostic for integration and hybridizations with both genomic Southern blots and pulsed-field gel electrophoresis blots according to standard procedures (Sambrook *et al*., 1989).

### Northern and Western blotting

RNA was extracted from purified 10-day-old stage V gametocytes using Trizol (Invitrogen) and analysed by Northern blot using BrightStar-Plus nylon membrane (Ambion). DNA probes (*Pfs16* and *RESA*) were amplified by PCR as previously reported (Sharp *et al*., 2006; Fivelman *et al*., 2007). Schizont and gametocyte protein preparations were boiled in reducing sample buffer, run on a 10% polyacrylamide gel and transferred to nitrocellulose membrane. Membranes were blocked in 2% ECL Advance (GE/Amersham Biosciences). Polyclonal antipeptide antibodies against the PfGK peptide CAKYNNNDIQKKTGTY were raised in rabbits and purified (Eurogentech). Blots were probed with the anti-PfGK primary antibody (1:500) and HRP-conjugated goat anti-rabbit secondary antibody (Bio-Rad; 1:20 000). Signals were detected using ECL reagents (GE/Amersham Biosciences).

### Microarray data

Normalized absolute expression values from *P. falciparum* microarray data from our recent analysis (Young *et al*., 2005) were plotted for: early rings, early trophozoites, early schizonts, merozoites, gametocyte stages II, III, IV and V and sporozoites.

### Construction of GK knockout plasmid

A 1342 bp 5′ flanking region of the *PfGK* gene and a 3′ flanking region of 1506 bp were inserted into the pHTK vector to mediate gene knockout. This vector contains the human dihydrofolate reductase (h*dhfr*) gene that confers resistance to the antifolate WR99210 for positive selection and the *Herpes simplex* thymidine kinase gene for negative selection. The two flanking fragments were inserted into the vector at either end of the *hdhfr* cassette to facilitate gene replacement. The fragments were amplified using the primer pairs 5′KOF-SpeI GGACTAGTCCTATATTATACACATCAATCATAC with 5′KOR-EcoRI GGGAATTCCTTAACATCGGATATGGGTA and 3′KOF-BglII GGAGATCTTTATCCTATAACTGCAGTACCATCATC with 3′KOR-ClaI GGATCGATTATTACACACAGTTCTTCAACC respectively (restriction sites underlined).

The primers used in diagnostic PCR for analysing the genotype of GK knockout clones were the *P. falciparum* GK upstream and downstream primers GKupF GTTTTGATTGACACACTTATTGTA and GKdnR CAATATATGTTTTCCTCATAGCATG. A double cross-over was confirmed with these primers to show a change in the resultant product size caused by replacement of the *GK* sequence with *DHFR*. For this PCR, an extended elongation time of 8 min was used.

### GFP reporter analysis

A stable episomally transformed *P. falciparum* line was produced comprising the 5′ untranslated region of PfGK upstream of the green fluorescent protein (GFP). Fluorescence microscopy was carried out in conjunction with sex-specific antisera to investigate GK expression in male and female gametocytes. Procedures are described in the legend to Fig. S1.

### Expression and purification of PfGK

The coding sequence of PfGK was amplified by PCR and cloned into the pMALc2x vector (New England Biolabs) using the BamHI and HindIII restriction sites. Expression in *E. coli* BL21* (Stratagene) produced PfGK as an MBP fusion with a Factor Xa cleavage site at its N-terminus. Overnight cultures grown in LB medium + 100 μg ml^−1^ ampicillin were used to inoculate autoinduction medium (Studier, 2005) at a ratio of 1:1000. The cultures were incubated for 8 h at 37°C and then 16°C overnight.

The cells were harvested by centrifugation and resuspended in buffer *A* (50 mM Tris pH 8.0 containing 50 mM arginine and 50 mM glutamate). Cell extract was loaded onto amylose resin high flow (New England Biolabs), equilibrated with buffer *A*, and the fusion protein was eluted in buffer *A* containing 10 mM maltose. Fractions containing PfGK–MBP were pooled for anion exchange chromatography using Q-Sepharose. Two distinct forms were eluted by a salt gradient that presumably reflect different oligomeric states, one of which was prone to aggregation. Fractions containing soluble PfGK–MBP were pooled for removal of MBP by proteolysis. Digestion with Factor Xa was incomplete even after prolonged incubation. Limited digestion with trypsin (1:100 ratio, 90 min at 20°C) revealed that this protease preferentially cleaves close to the Xa site. The Mr of cleaved PfGK was measured by mass spectrometry to be 57 289, which corresponds to the expected mass of the 501 PfGK residues plus an additional six N-terminal residues from the linker region (calculated Mr 57 273). The digestion products were loaded onto a Superdex 200 gel filtration column equilibrated with 50 mM Tris pH 7.5 and 50 mM NaCl. PfGK eluted as a peak with a retention volume corresponding to a Mr of 115 000 implying a dimer.

### Native GK activity assays

Purified stage V gametocytes and mature asexual schizonts were assayed for cellular GK activity using [^3^H]-glycerol (TRA-118, GE/Amersham Biosciences) (Guan *et al*., 2002). Activity was measured in the form of counts per minute incorporated into [^3^H]-G3P. Aliquots of 10^7^ purified cells were re-suspended to a final volume of 100 μl with lysis buffer (25 mM Tris HCl pH 7.0, 15 mM MgCl_2_, 2 mM MnCl_2_, 1 mM DTT, 10 μM NaF and Mammalian Tissue protease inhibitors) before storage at −80°C. Parasites were lysed by three rounds of freeze–thaw in a dry ice/ethanol bath followed by incubation at 30°C before being centrifuged at 12 000 *g* for 5 min to remove membrane fractions. Five microlitres of the resulting cytoplasmic fractions were incubated in a 50 μl reaction volume [25 mM Tris HCl (pH 7.0), 15 mM MgCl_2_, 2 mM MnCl_2_, 1 mM DTT, 45 μM ATP, 10 μM β-glycerol-2-phosphate, 10 μM NaF and 160 μM [^3^H]-glycerol] at 30°C for 30 min. The reaction was halted by the addition of 100 μl methanol. 30 μl volumes were spotted onto DE81 paper (Whatman, UK), washed in H_2_O and dried before addition of 3 ml of scintillation fluid (Optiphase ‘Supermix’, PerkinElmer). Radioactivity was measured using a Beckman LS6000LL scintillation counter. Background activity of a control reaction with H_2_O added instead of parasite extract was subtracted. For details of derivation of kinetic constants, see legend to Fig. S3.

### Crystallization and structure solution

PfGK (3.5 mg ml^−1^ in 50 mM Tris pH 7.5, 50 mM NaCl) was crystallized by vapour diffusion. Hanging drops consisting of 1 μl protein and 1 μl of reservoir solution containing 50 mM potassium formate, 20% PEG 3350, 25% ethylene glycol, 10 mM lauryldimethylamine oxide and 20 mM glycerol (Solution A) were equilibrated against 0.5 ml reservoir solution at 20°C. The resulting thin crystal plates were used as seeds to obtain larger crystals. For ADP complex formation, crystals were soaked for 4 days in solution A containing 5 mM ADP and 10 mM MgCl_2_. Although the appearance of the PfGK–ADP and PfGK-glycerol crystals is similar, the cell dimensions and molecular packing are different. It is therefore likely that the crystals dissolved and regrew during the period of soaking although these experiments were not monitored closely.

Data were collected on synchrotron beamline ID14.2 at the ESRF and analysed using programs in the CCP4 suite (CCP4, 1994). Data collection and refinement statistics are summarized in Table 1. For the PfGK–glycerol data set, all data were used in molecular replacement calculations in the program molrep (Vagin and Teplyakov, 2000) using EcGK (PDB code 1bu6) as a search model (41% sequence identity). Automated model building was carried out using ARP/wARP (Perrakis *et al*., 1999). After six cycles of autobuilding, 1769 residues were found with sequence coverage of 92%. During this process the *R*-factor dropped from 0.543 (*R*_free_ 0.539) to 0.233 (0.277). The model was refined by iterative cycles of refmac (Murshudov *et al*., 1997) using anisotropic temperature factors. Manual modelling of missing fragments and final adjustments were carried out in COOT (Emsley and Cowtan, 2004). Refinement was concluded with an *R*-factor of 0.15 (*R*_free_ 0.20) for all data and a final model containing all 501 residues of four chains from two dimers, 12 glycerol molecues (four in the active sites), 62 ethylene glycol molecules (present at 25% in the crystal cryoprotectant) and 1929 water molecules. These protein chains can be superimposed to give pairwise root mean squared deviations in Cα positions in the range 0.2–0.3 Å. Superposition onto chains of the EcGK molecular replacement model gives root mean squared deviation values of ∼1.5 Å. Interestingly, PfGK contains 12 cysteine residues, none of which are conserved in EcGK and none of which are involved in disulphide bond formation, although the sulphur atoms of Cys251 and Cys292 are separated by only 4 Å.

The PfGK–glycerol dimer model was used to find a molecular replacement solution for the PfGK–ADP data set. The MOLREP solution was unsatisfactory, with only a single domain of each monomer occupying electron density. The model was partitioned into separate domains. Domains I (residues 1–307 and 440–471) were fixed while a search was carried out with domains II comprising the remainder of the model. This produced a clear solution with the resulting domains II shifted by symmetry operations and connected to the corresponding domains I, revealing one dimer in the asymmetric unit. The model was refined by iterative cycles of refmac and manual modelling in COOT. TLS parameterization and NCS restraints applied towards the end of refinement improved the statistics with a resulting *R*-factor of 0.19 (*R*_free_ 0.25). The final model contains two protein chains, two ADP ligands and 21 ethylene glycol species, four of which are located in the two glycerol binding pockets. Ethylene glycol is present at 200 times the concentration of glycerol after addition of the cryoprotectant to the crystal. The reason why ethylene glycol displaces glycerol in the open PfGK–ADP complex but not the closed PfGK–glycerol complex is not clear, although it may be due to the longer time of incubation of the PfGK–ADP crystals in the cryoprotectant solution prior to freezing.

Ramachandran plots of the glycerol-and ADP-liganded models according to PROCHECK show that 92.3% and 88% of the residues, respectively, lie in the most favoured region. Residues Ala312 and Tyr354 of both structures fall into the generously allowed region. Glu84, which is involved in glycerol binding, is the only residue that lies in a disallowed region of the Ramachandran plot in all molecules, an observation that is notable as steric strain in well-refined structures correlates with functional significance (Herzberg and Moult, 1991).

### Data deposition

The co-ordinates and structure factors for PfGK–glycerol (pdb code 2w40) and PfGK–ADP (pdb code 2w41) have been deposited in the Protein Data Bank (http://www.pdb.org).
