# Supplementary material for: Structure and non-essential function of glycerol kinase in Plasmodium falciparum blood stages
Source: Mol Microbiol. 2009 Jan;71(2):533–45. doi: 10.1111/j.1365-2958.2008.06544.x (PMC2680290; doi:10.1111/j.1365-2958.2008.06544.x)
Supplement: Supplementary file 1 [file mmi0071-0533-SD1.pdf]

Supplementary Material

Structure and non-essential function of glycerol kinase in *Plasmodium falciparum* blood stages

Claudia Schnick, Spencer D. Polley, Quinton L. Fivelman, Lisa C. Ranford-Cartwright,
Shane R. Wilkinson, James A. Brannigan, Anthony J. Wilkinson and David A. Baker

Fig. S1

Immunofluorescence analysis of *P. falciparum* parasites stably transfected with episomes expressing GFP under the control of the PfGK 5' untranslated sequence.

Fig. S2

Functional complementation of *E. coli* GK^{-} mutants.

Fig. S3

Glycerol kinase activity assays.

Fig. S4

Regulation of glycerol kinase activity.

Movie.mp4

This file was created in CCP4mg (Potterton *et al.*, 2004) by Liz Potterton (University of York, UK). It is a movie that will play in a web browser and shows the 27° domain movement (Hayward & Lee, 2002) between open and closed forms.

References for Supplementary Material

Figure S1. Immunofluorescence analysis of *P. falciparum* parasites stably transfected with episomes expressing GFP under the control of the PfGK 5' untranslated sequence.

GFP expression was visualized with UV light and appears green in the micrographs. Nuclear staining with DAPI appears blue. Parasites in panels in **(A)** have been stained with a female gametocyte-specific antibody and **(B)** with a male gametocyte-specific antibody and both appear red. Size bars indicate 10 µm. The transfectants show evidence of GK promoter activity in both male and female gametocytes.

(A)

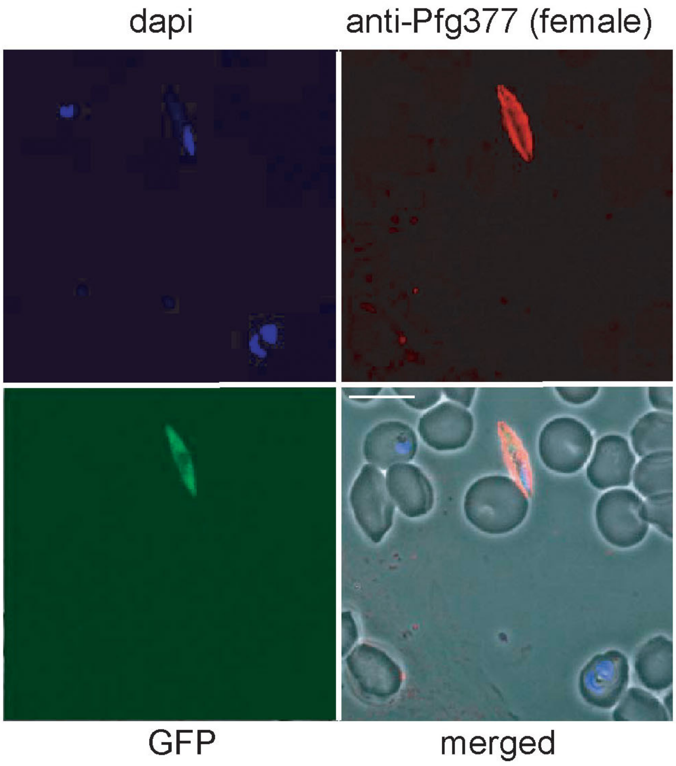

(B)

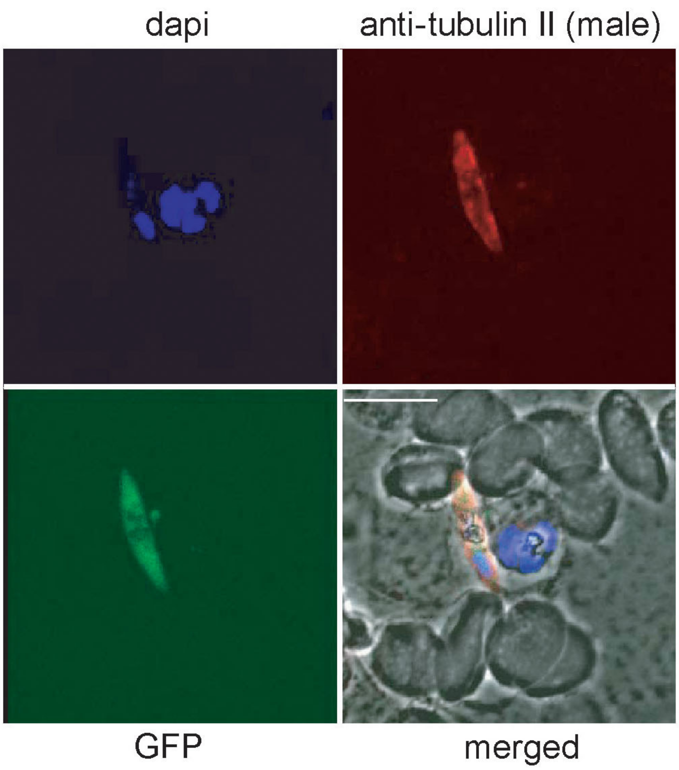

GFP reporter analysis.

A stable episomally-transformed *P. falciparum* line was produced comprising the 5' untranslated region of PfGK upstream of the green fluorescent protein (GFP). The Gateway plasmid pHGB was used as the entry vector (kindly sent to us by Christian Doerig, University of Glasgow and was made by Chris Tonkin, Melbourne, Australia). A 1.6 kb upstream region to the putative glycerol kinase was amplified using the primers gcggtcgacTAATACATCACGTTGTTGTTATT and gcctaggACTTAATATGACATTCATTTTATTTA (Sigma-Genosys; *Sal*I and *Avr*II sites underlined, respectively) and ligated into the *Sal*I and *Avr*II sites of pHGB replacing the PfHSP86 promoter. To produce the final transfection vector, Gateway LR clonase reactions were performed according to the manufacturer's instructions (Invitrogen) using the pCHD-1/2 destination vector that contains a hDHFR for drug selection (Tonkin *et al.*, 2004). Resulting plasmids were checked for correct recombination by restriction digestion and sequence analysis, and purified using a Qiagen MegaPrep kit. Transfection and positive selection using WR99210 was performed as described by Duraisingh and colleagues (Duraisingh *et al.*, 2002).

Parasite preparations were washed twice in Phosphate Buffer Saline (PBS) solution containing 4 % (w/v) paraformaldehyde, resuspended to a 20 % haematocrit and spotted onto wells on IFA slides (Hendley, Essex). Dried slides were fixed in acetone on ice for 10 min, washed in PBS, and blocked in PBS-0.1% BSA for 30 min at 22°C in the dark. Primary antibodies rabbit anti-alpha tubulin II (from MR4; http://www.mr4.org/) and rat anti-PfG377 (a kind gift from Pietro Alano) both diluted 1:250 were applied in PBS-0.1% BSA for 1 h, the slides were then washed and rhodamine-conjugated fluorescence-labeled secondary antibodies (donkey anti-rabbit immunoglobulin G, Jackson Immunoresearch Laboratories, USA and goat anti-Rat immunoglobulin G, Pierce, Rockford, USA) both diluted 1:400 were added for 1 h, respectively. The parasites were then given a final wash in PBS, the slides mounted in Vectashield containing nucleic acid stain DAPI (Vector Laboratories, USA), and stored in the dark at 4ºC until visualized using Zeiss confocal fluorescence microscopy.

Figure S2. Functional complementation of *E. coli* GK^{-} mutants.

PfGK fused to *MBP* in the pMAL plasmid was tested for its ability to complement *E. coli* GK^{-} mutants using McConkey agar medium following a method based on that described by Guo and colleagues (Guo *et al.*, 1993). *E. coli* HB101 (5 µl of overnight cultures) containing a Tn5 insertion in the *glpK* gene (strains Tn5-6, 9, 17, 18, 20, 21 obtained from E. McCabe), transformed with WT or recombinant plasmids (or untransformed) were spotted onto grid-marked McConkey plates supplemented with 1 % glycerol, 50 µg/ml kanamycin, 100 µg/ml ampicillin and 100 µM IPTG and grown at 28°C overnight.

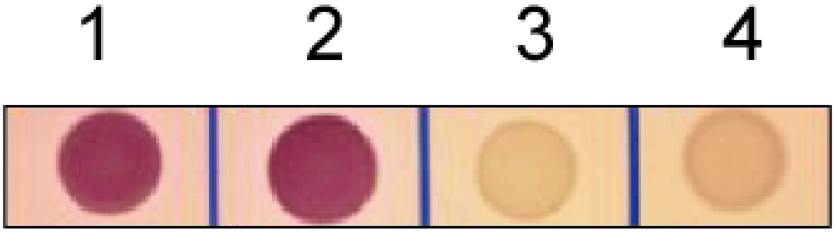

A section of a McConkey agar plate supplemented with 1 % glycerol and 100 µM IPTG. Two independent *E. coli* HB101 Tn5 GK-deficient mutants (Tn5-20 and Tn5-21) are shown either transformed (1 and 2) with *PfGK* in the pMAL-C2X plasmid or untransformed (3 and 4). Overnight cultures (2.5 µl) were spotted on to the plate and grown for 16 hours at 28°C. Purple colonies indicate that the bacteria can utilise glycerol as a carbon source whereas yellow colonies indicate the converse.

Figure S3. Glycerol kinase activity assays.

(A) ***Scheme for coupled reactions.***

Forward reaction:

Glycerol + ATP ⇌ (*Glycerol kinase*) Glycerol-3-phosphate + ADP

ADP + Phosphoenolpyruvate ⇌ (*Pyruvate kinase*) ATP + Pyruvate

Pyruvate + NADH + H^+ ⇌ (*Lactate dehydrogenase*) Lactate + NAD^+

(B) ***V_{max} and K_m kinetic curves for the reaction with glycerol and ATP.***

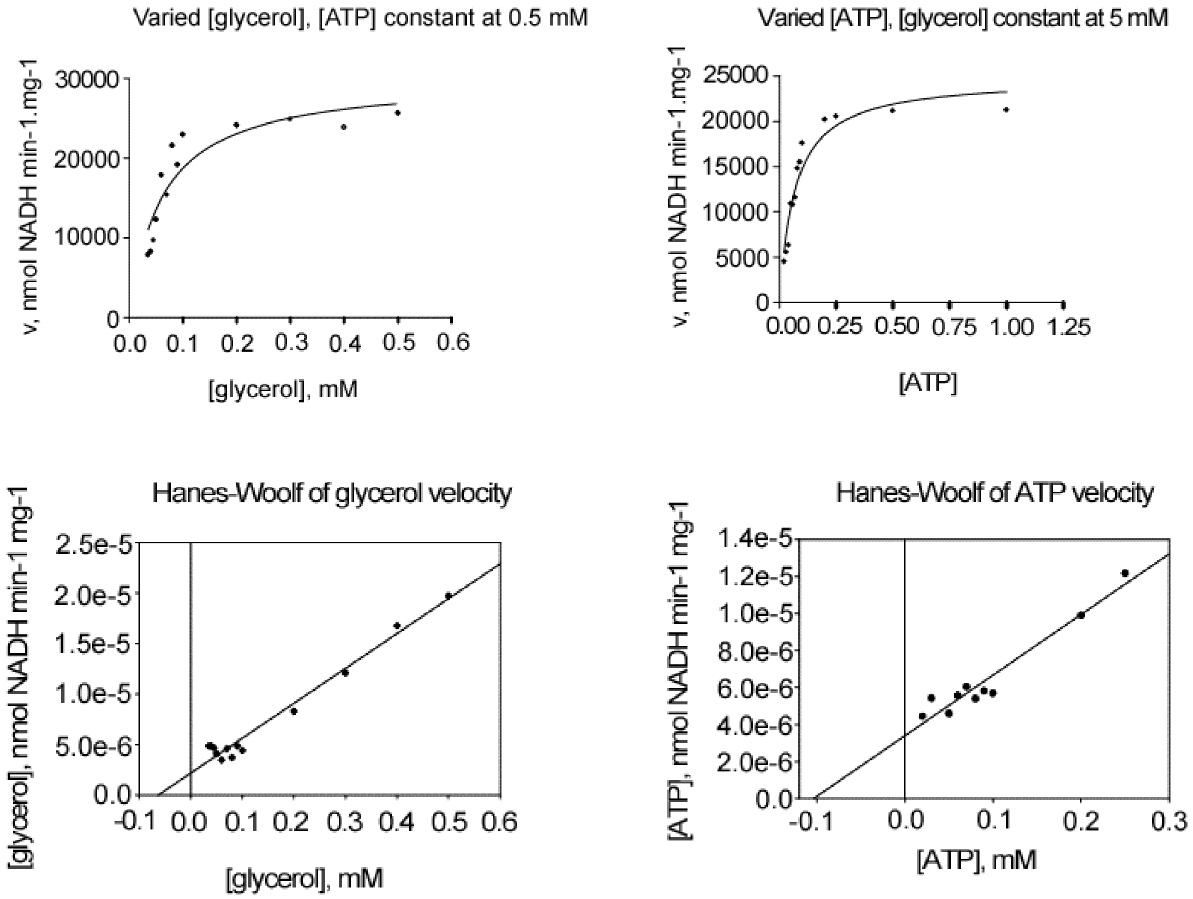

Derivation of kinetic constants.

The phosphotransferase activity of the purified PfGK was measured indirectly by spectrophotometry in a coupled system with pyruvate kinase and lactate dehydrogenase as auxiliary enzymes (Kralova *et al.*, 2000). The reverse thermodynamically unfavourable reaction of PfGK was, as anticipated, too slow for accurate kinetic parameters to be derived.

The forward reaction was followed by measuring the decrease in NADH absorbance at 340 nm ($\varepsilon = 6.22$ mM^{-1} cm^{-1}) in a system coupled with pyruvate kinase and lactate dehydrogenase (see SI Fig.3). The reactions were carried out at 25°C in a volume of 1 ml containing 120 mM Tris pH 8.0, 0.5 mM EDTA, 10 mM $MgCl_2$, 100 mM KCl. The final concentrations of the reactants were as follows: 0.2 mM NADH, 5 mM phosphoenolpyruvate, 5 mM glycerol, 2.5 mM ATP. Pyruvate kinase (Fluka) and lactate dehydrogenase (Sigma) were used at 12 U/ml. The reaction mix was preincubated for 1 minute at 25°C and reactions were started by adding purified glycerol kinase at 1 µg/ml (0.0176 µM). The reaction was monitored continuously over 120 s. The linear part of the reaction curve was used to calculate the initial rate of substrate conversion with NADH decreasing in an equimolar ratio to the ATP converted by glycerol kinase. K_M and V_{max} for ATP were determined with glycerol at a constant concentration of 5 mM and ATP in a range between 2.5 µM and 339 µM. K_m and V_{max} for glycerol were measured at a constant ATP concentration of 2.5 mM and glycerol varying between 2.5 and 300 µM.

Figure S4. Regulation of glycerol kinase activity.

(A) **(B)**

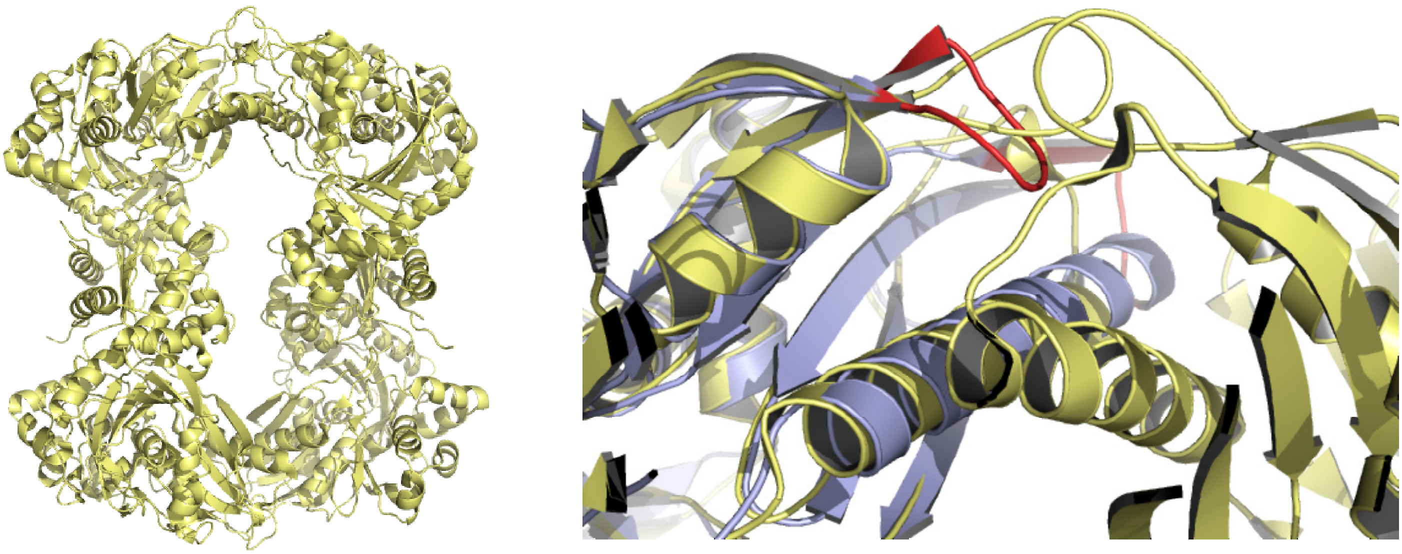

(A) Tetramer of *E. coli* GK. **(B)** The interface loop 227-238, which is involved in EcGK tetramer formation superposed with PfGK. In EcGK (yellow) the loop rises upwards from the dimer core and it is able to bind FBP on the 2-fold axis. In contrast, the corresponding loop in PfGK (red) is projects downwards and does not form inter-chain connections.

References for Supplementary Material

Duraisingh, M. T., T. Triglia & A. F. Cowman, (2002) Negative selection of Plasmodium falciparum reveals targeted gene deletion by double crossover recombination. *International Journal for Parasitology* **32**: 81-89.

Guo, W. W., K. Worley, V. Adams, J. Mason, D. Sylvesterjackson, Y. H. Zhang, J. A. Towbin, D. D. Fogt, S. Madu, D. A. Wheeler & E. R. B. McCabe, (1993) Genomic scanning for expressed sequences in XP21 identifies the glycerol kinase gene. *Nature Genetics* **4**: 367-372.

Hayward, S. & R. A. Lee, (2002) Improvements in the analysis of domain motions in proteins from conformational change: DynDom version 1.50. *Journal of Molecular Graphics & Modelling* **21**: 181-183.

Kralova, I., D. J. Rigden, F. R. Opperdoes & P. A. M. Michels, (2000) Glycerol kinase of Trypanosoma brucei - Cloning, molecular characterization and mutagenesis. *European Journal of Biochemistry* **267**: 2323-2333.

Potterton, L., S. McNicholas, E. Krissinel, J. Gruber, K. Cowtan, P. Emsley, G. N. Murshudov, S. Cohen, A. Perrakis & M. Noble, (2004) Developments in the CCP4 molecular-graphics project. *Acta Crystallographica Section D-Biological Crystallography* **60**: 2288-2294.

Tonkin, C. J., G. G. van Dooren, T. P. Spurck, N. S. Struck, R. T. Good, E. Handman, A. F. Cowman & G. I. McFadden, (2004) Localization of organellar proteins in Plasmodium falciparum using a novel set of transfection vectors and a new immunofluorescence fixation method. *Molecular and Biochemical Parasitology* **137**: 13-21.
