# Supplementary figures and images for: Structure and non-essential function of glycerol kinase in Plasmodium falciparum blood stages
Source: Mol Microbiol. 2009 Jan;71(2):533–45. doi: 10.1111/j.1365-2958.2008.06544.x (PMC2680290; doi:10.1111/j.1365-2958.2008.06544.x)

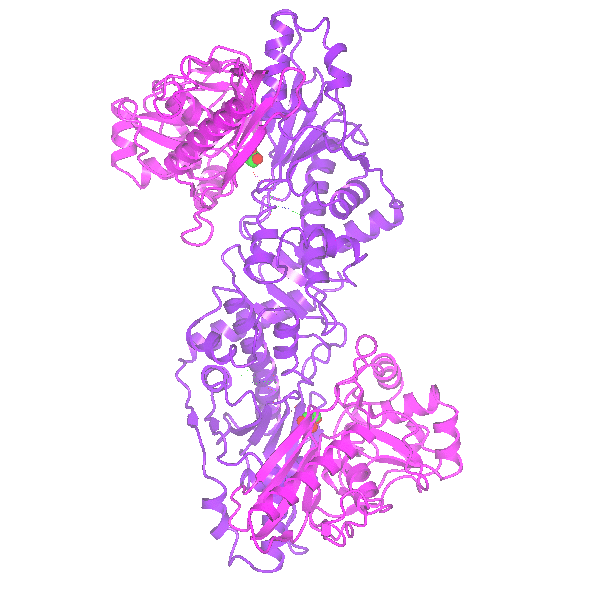

Supplement: Supplementary file 2 [file mmi0071-0533-SD2.gif]
